# The XPA Protein—Life under Precise Control

**DOI:** 10.3390/cells11233723

**Published:** 2022-11-22

**Authors:** Yuliya S. Krasikova, Olga I. Lavrik, Nadejda I. Rechkunova

**Affiliations:** 1 Institute of Chemical Biology and Fundamental Medicine, Siberian Branch of Russian Academy of Sciences, Novosibirsk 630090, Russia; 2 Department of Natural Sciences, Novosibirsk State University, Novosibirsk 630090, Russia

**Keywords:** XPA, nucleotide excision repair (NER), DNA repair, post-translational modifications, PARP1, PARylation, ATR, phosphorylation

## Abstract

Nucleotide excision repair (NER) is a central DNA repair pathway responsible for removing a wide variety of DNA-distorting lesions from the genome. The highly choreographed cascade of core NER reactions requires more than 30 polypeptides. The xeroderma pigmentosum group A (XPA) protein plays an essential role in the NER process. XPA interacts with almost all NER participants and organizes the correct NER repair complex. In the absence of XPA’s scaffolding function, no repair process occurs. In this review, we briefly summarize our current knowledge about the XPA protein structure and analyze the formation of contact with its protein partners during NER complex assembling. We focus on different ways of regulation of the XPA protein’s activity and expression and pay special attention to the network of post-translational modifications. We also discuss the data that is not in line with the currently accepted hypothesis about the functioning of the XPA protein.

## 1. Introduction

The nucleotide excision repair (NER) pathway is the most universal repair pathway for the removal of a wide range of structurally unrelated DNA lesions, including UV photolesions (e.g., cyclobutane pyrimidine dimers (CPDs) and pyrimidine-pyrimidone (6-4)-photoproducts (6-4PPs)), intrastrand crosslinks, reactive oxygen species-induced base alterations, and bulky adducts of DNA bases with reactive metabolites of some chemical carcinogens or chemotherapeutic agents [1,2,3]. Mutations in NER-related genes are associated with an autosomal recessive disease called xeroderma pigmentosum (XP) [4]. XP is characterized by extreme sensitivity of the skin to sunlight and a dramatically increased risk of skin cancer [5,6]. A subset of XP patients developed a profound neurodegenerative condition known as XP neurological disease [7]. XP patients can be classified into seven complementation groups, XP-A through XP-G, depending on the specific gene that is affected [1]. Patients with known mutations in the *XPA* gene have the most severe form of XP, indicating a critical role of the XPA protein in the NER process.

Initially, XPA was considered to be the sole damage recognition factor [8]; then, it was proposed that the XPA–RPA complex performs the first recognition [9,10]. Later, enough data was accumulated to suggest that lesions are primarily recognized by the XPC–RAD23B–CEN2 complex [3], and XPA takes part in the damage verification process (together with TFIIH) and overall plays the role of an organizing or scaffold component of NER [11,12,13].

Three-dimensional structures of XPA give us an opportunity to propose a spatial arrangement of XPA inside NER machinery. Today, several spatial structures of human XPA exist: two solution structures of the minimal DNA-binding domain (DBD) determined by NMR spectroscopy (Protein Data Bank (PDB) IDs 1XPA and 1D4U) [14,15], a crystal structure of a redefined XPA DBD (PDB ID: 6J44), and a crystal structure of the XPA–DNA complex (PDB ID: 6LAE) [16,17]. The series of crystal structures of a yeast homolog of XPA (Rad14) in a complex with different lesion-containing DNA substrates illustrates the DNA-binding capacity of the XPA DBD [18,19,20]. Recent advances in cryo-electron microscopy (cryo-EM) gave investigators a unique chance to look into the structure of a TFIIH–XPA–DNA complex (PDB: 6RO4) [21] and confirm the biochemical data about XPA localization inside the DNA repair bubble [22]. Furthermore, cryo-EM data have expanded our knowledge about the modulation of TFIIH activity by XPA and XPG.

This review provides updated information and additional insights into the XPA structural features and the XPA protein’s interaction partners in light of the present knowledge about the contact surface on XPA. We also briefly describe the NER assemble from the standpoint of XPA’s organizing role. Then, we summarize data on the mechanisms of the *XPA* gene transcription control, and finally, we describe the regulatory mechanisms of XPA post-translational modifications (PTMs) and combine them into a unified network.

## 2. XPA’s Structure and DNA-Binding and Protein-Binding Abilities

**Figure 1:**
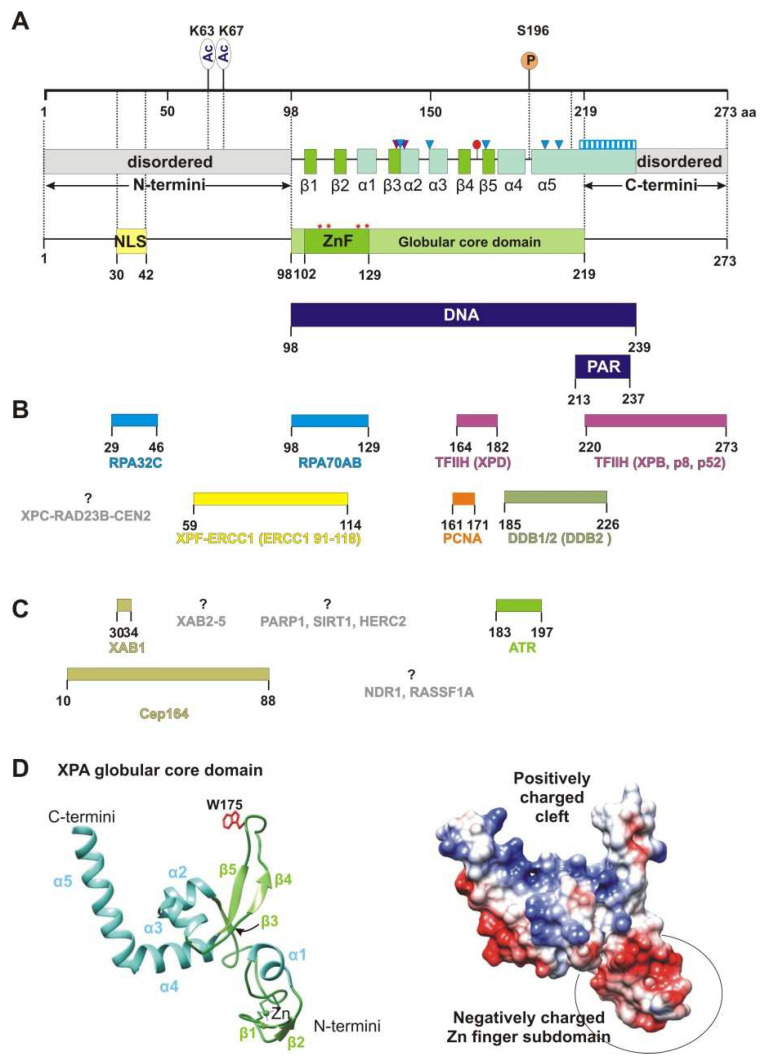
XPA’s structure and interaction partners. (**A**) The map of the XPA domain structure and known points of PTMs: phosphorylation at S196 and acetylation at K63 and K67. Secondary-structure elements are shown according to crystal structures PDB 6LAE and 6J44: β-strands are green (β1: aa 103–104, β2: aa 111–112, β3: aa 138–140, β4: aa 164–167, and β5: aa 178–172), and α-helices are light blue (α1: aa 116–121, α2: aa 141–148, α3: aa 151–157, α4: aa 183–194, and α5: aa 197–239). Positively charged residues K141, K151, K179, R207, and R211, which are directly involved in interactions with backbones of a DNA duplex, are shown as blue triangles. Two residues (Thr140 and Thr142, indicated as purple triangles) interact with the DNA backbone through a van der Waals contact and a hydrogen bond, respectively. Extended helix α5 contains several positively charged residues (Lys217/218/221/222/224/236 and Arg227/228/231/237) that are possibly involved in DNA binding, which are shown as a blue striped box. Conserved residue Trp175 intercalates into unpaired bases of single-stranded DNA (ssDNA) at the ss–dsDNA junction and is displayed as a red circle. Unstructured N- and C-terminal regions are gray. Zinc-coordinated conserved cysteine residues (C105, C108, C126, and C129) are presented as red asterisks and a Zn-finger motif (ZnF, aa 102–129) colored green. The N terminus accommodates a nuclear localization signal (NLS, aa 30–42), which is yellow. DNA-binding (aa 98–239) and poly(ADP-ribose) (PAR)-binding (aa 213–237) motifs are mapped to the overall XPA structure and are highlighted in dark blue. (**B**) Interaction sites for NER protein partners on XPA, which are aligned with the XPA residues involved in each interaction. Proteins whose interaction sites are unknown are gray. (**C**) XPA interaction partners outside NER. (**D**) A structural model of the XPA globular core domain (PDB ID: 6LAE). A ribbon diagram with color codes according to (**A**). The Trp175 residue is shown in red. A distribution of the electrostatic potential on the surface for the same structure: a positive charge is shown in blue, and a negative charge is red. The structures were generated using UCSF Chimera software (version 1.16).

XPA is one of the smallest proteins inside the NER machine: human XPA is 31 kDa and consists of only 273 amino acid residues (aa). XPA is composed of a central globular domain (aa 98–219) that is flanked by dynamically disordered N and C termini (Figure 1A). This kind of structural organization, when structured globular domains are combined with disordered regions, is very common among eukaryotic proteins [23]. Frequently, disordered proteins (entirely disordered or containing disordered sequences, as in XPA) interact with or function as hubs in protein interaction networks or play a central role in an ordered assembly of macromolecular machines [24,25]. Indeed, both low-sequence-complexity parts enable the XPA protein to interact with a variety of protein partners. Future experiments will shed light on disordered XPA part properties: do they do folding upon binding to protein partners (and should be called “intrinsically disordered”), or do these regions not adopt a specific three-dimensional structure during functioning. In addition, the N terminus contains a conserved nuclear localization signal (NLS) that lies in the 13-residue stretch from aa 30 to 42 [26,27] (Figure 1A). The NLS is a tag that ensures that the protein is sorted into the nucleus, but in the case of XPA, things are not so simple, and we discuss this matter below.

The central domain contains a C4-type Zn-finger (ZnF) motif that has the sequence Cys_105_-X_2_-Cys_108_-X_17_-Cys_126_-X_2_-Cys_129_ [28] (Figure 1A). Side chains of cysteines Cys105/108 and Cys126/129 coordinate the zinc ion [14]. Although the ZnF of XPA is essential for DNA binding and for NER activity [29], the ZnF core itself is not directly in contact with DNA [16] but rather properly ensures the DBD folding [30]. Moreover, the zinc-containing subdomain is even negatively charged due to many glutamate and aspartate residues [14] (Figure 1D). Notably, the UvrA protein has two units of the same C4 type of the ZnF motif [28].

Originally, the minimal DNA-binding domain (DBD) of XPA was mapped to a central globular core between residues 98 and 219 [14,15,29,31,32]. This region contains a sheet-helix-hairpin motif (residues 138–182) and a helix-turn-helix motif (residues 183–230) that form a shallow clamp-like or right hand-like structure (the sheet-helix-hairpin motif as fingers and helix-turn-helix as a thumb) with a positively charged inner surface (Figure 1D). The internal curvature of the basic cleft fits well to the diameter of a standard B-form dsDNA [14]. Subsequent studies have found that some residues on the C-terminal side beyond the minimal DBD domain are also involved in binding to DNA substrates [33,34,35]. Later, the XPA DBD was redefined and extended by 20 additional C-terminal residues (Asp220–Thr239). The redefined XPA DBD (aa 98–239) can bind to DNA with an affinity nearly identical to that of the full-length XPA protein [34,35]. The crystal structure revealed that the C-terminal extension folds as a long α-helix (α5 in Figure 1A) with a basic residue cluster resulting in the formation of a consecutive positively charged surface [16,17]. Interestingly, structural superposition of the human XPA DBD on a yeast Rad14–DNA complex implies that the α5 extension (Asp217–Thr239) cannot directly come into contact with a DNA substrate.

**Figure 2:**
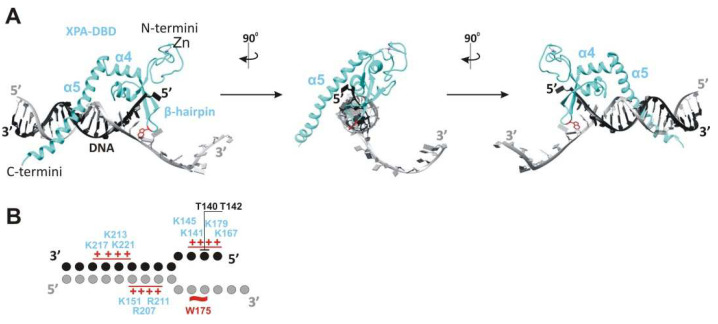
XPA interaction with the ss–dsDNA junction. (**A**) Cryo-EM structure PDB: 6RO4 provides details of the XPA–DBD interaction with the ss–dsDNA junction. XPA demarcates the 5′ edge of the DNA repair bubble. XPA inserts its intercalating β-hairpin between DNA single strands at the junction. Red colored Trp175 from the tip of the β-hairpin stacks against the base of the DNA 3′-extension at the junction. The structures were generated using UCSF Chimera software (version 1.16). (**B**) Schematic representation of the interactions between side chains of the XPA and DNA junction, according to cryo-EM structure PDB: 6RO4 [21] and crystal structure PDB: 6LAE. DNA nucleotides are indicated as circles. Patches of positively charged residues in proximity to the DNA backbone are indicated by red pluses. Hydrogen bonding of T142 and a van der Waals contact of T140 are indicated as black lines.

XPA has an ability to recognize some bulky lesions [22,36] and especially prefers to bind to kinked and branched DNA structures [37,38,39]. All of these DNA structures contain an ss–dsDNA junction. XPA binds to the duplex part of the junction in a non-sequence-specific manner via electrostatic interactions between the positively charged cleft and negatively charged phosphate backbones of the DNA duplex [17] (Figure 1F and Figure 2). This intermediate is further stabilized by hydrogen bonding of the side chain hydroxyl group of Thr142, and a van der Waals contact is formed between the side chain Cβ atom of Thr140 and DNA’s phosphate moieties (Figure 1A and Figure 2). Thereafter, Trp175 from the top of the hairpin between β4 and β5 is stacked with bases of the unpaired ssDNA at the junction, thereby giving rise to a stable conformation of this β-hairpin [17,21]. According to the energy calculations, binding to ssDNA in the 3′→5′ direction is more favorable than that in the 5′→3′ direction (relative to α5), but it is not observed experimentally [29,38].

Recently, it was shown with atomic force microscopy, scanning force microscopy, and mathematical modeling that XPA undergoes episodic one-dimensional diffusion to search the DNA for damage [40]. The functional meaning of XPA’s damage recognition ability is not clear, and today, XPA is considered only as a protein scaffold element inside the NER complex. Anyway, XPA interacts with the proteins involved in every step of NER, from damage recognition to DNA synthesis. Yet, it is unknown how many contacts XPA could engage in concurrently, and it is possible that XPA interacts with these proteins not simultaneously but in the order of them joining the repair machinery. Figure 1B lists XPA’s protein interaction partners directly involved in NER according to the following process steps:

### 2.1. Initial Damage Recognition

XPA interacts physically with DDB2 through aa 185–226, and this interaction can be seen both in vitro and in vivo [41]. UV-damaged DNA-binding protein (DDB1/2) is a heterodimeric protein consisting of subunits DDB1 and DDB2/XPE and has an extraordinarily high binding affinity and specificity for CPD and 6-4PP [2,3]. The biological role of the XPA–DDB2 interaction is unclear, but because the DDB2 interaction site overlaps with the poly(ADP-ribose) [PAR]-binding motif (aa 213–237), we can speculate that the XPA–DDB2 complex is involved in PAR-dependent chromatin remodeling together with PARP1 and XPC [42]. XPC (which functions in the complex with proteins RAD23B and CEN2) is the protein sensor responsible for the detection of a wide variety of DNA lesions that are repaired through the global genome NER (GG-NER) pathway [43]. XPA interacts with XPC and stimulates its binding to the DNA [44,45]. The XPC interaction site in the XPA sequence and the biological meaning of this interaction are unknown.

### 2.2. Damage Verification

The TFIIH complex is the key protein for the damage verification step. Depending on the context, the TFIIH composition changes from a core of seven subunits, including translocase XPB and helicase XPD, to ten subunits, through the addition of three CAK (Cdk-activating kinase module) kinase subunits [21,46]. XPA’s whole C terminus is involved in an interaction with the TFIIH protein [47]. Recent cryo-EM data expanded our knowledge about the TFIIH–XPA interaction [21]. XPA (together with nuclease XPG) facilitates the CAK kinase module release from the core TFIIH and stabilizes an alternative conformation of TFIIH, where the XPD helicase assumes the open conformation for the functioning. In this complex, XPA forms a bridge between the XPB and the XPD, and moreover, XPA’s extended α5 helix and ATPase XPB form a positively charged tunnel that holds the DNA within. Thus, by trapping the DNA within a duplex tunnel, XPA may keep the NER machinery on the DNA during lesion scanning and processing [21].

### 2.3. Pre-Incision Complex Formation

Immediately after TFIIH opens the DNA repair bubble, the undamaged ssDNA that is being formed is bound by replication protein A (RPA) [48]. RPA is a heterotrimer consisting of subunits RPA70, RPA32, and RPA14 [49,50]. XPA interacts with two of them: RPA70 (which contains OB-fold domains A, B, and C) and RPA32 (which contains the D OB-fold domain) [51]. It was shown recently that the interaction between XPA (aa 29–46) and RPA32C is important for the initial association of XPA with NER complexes, whereas the interaction between XPA (aa 98–126) and RPA70AB is needed for structural shaping of the complex to enable the dual incision reaction [52]. Pre-incision complex formation is completed by the engagement of nuclease XPF–ERCC1, which is recruited by the XPA through the interaction with ERCC1 [53,54,55,56,57]. XPA aa 59–114 are responsible for this interaction. In particular, the Gly72–Phe75 region (also known as the G motif) and the Glu78–Glu84 region (i.e., the E motif) are the residues necessary for the binding of XPF–ERCC1. Biochemical data have shown that the ZnF motif (aa 102–129) is partially involved in this contact, but the NMR and molecular dynamics simulation revealed that only the 14-amino acid sequence (aa 67–80) mediates this interaction.

### 2.4. Dual Incision, Resynthesis, and Ligation

After the first incision, proliferating cell nuclear antigen (PCNA) joins the NER complex. PCNA is the processivity factor for DNA polymerases. XPA has been found to interact directly with PCNA via the APIM sequence (the AlkB homolog 2 PCNA-interacting motif), and it has been shown that XPA and PCNA colocalize to the damaged DNA foci in a cell culture [58,59,60]. XPA^−/−^ cells complemented with XPA containing a mutated APIM sequence have high UV sensitivity and a deficient repair of CPDs and 6-4PPs and are consequently more arrested in the S phase as compared to XPA^−/−^ cells complemented with wild-type XPA. Notably, XPA colocalizes with PCNA in the replication foci and is loaded onto a newly synthesized DNA in undamaged cells; thus, it is possible that this interaction is required for DNA-processing pathways other than NER.

### 2.5. XPA Dimerization

It is well-known that isolated XPA easily forms a homodimer. Moreover, in vitro, it can form the XPA_2_–RPA complex [61]. Instead, XPA has been widely assumed to be a monomer participating in the mechanism of NER (the first statement about the monomeric functional form was published by [62]. Accordingly, the physiological meaning of the XPA dimerization and the structural mechanism of this process are still unclear. Recently obtained molecular dynamics simulation data indicate that some residues make a contribution to the intermolecular interactions in XPA homodimers, but this needs to be validated by another approach [63]. Notably, XPA is not the sole NER protein that demonstrates easily dimerization characters, but as in the case of XPA, there is no functional explanation found for this ability [64].

### 2.6. PTM Proteins

Today, it is known that XPA is precisely tuned by several PTMs [13,65,66]. Obviously, XPA interacts with proteins that provide these modifications and removes them, but we know the interaction site only for the ATR kinase: the α4 helix [67] (Figure 1C). Interaction sites with proteins facilitating these modifications—RASSF1A and NDR1—have not been determined either. The only interaction site that has been identified is the one for the Cep164 protein, which functions in ATR-mediated checkpoint activation; it interacts with XPA through aa 10–88 of XPA [68].

### 2.7. XABs

Using the yeast two-hybrid system, researchers identified a novel set of XPA-interacting proteins that was designated as XABs [69]. XAB1 is a cytoplasmic protein with GTPase activity and binds to the N-terminal region (residues 1–52) of XPA, and the region “aa 30–34” is directly involved in this interaction (Figure 1C). The XAB1-interacting site overlaps with the NLS and raises a question about XAB1′s role in the cytoplasmic sequestering of XPA. Among the five found XABs, only XAB2 has a nuclear function and is intensively investigated. It has been reported that XAB2 interacts with the proteins involved in transcription-coupled NER (TC-NER), for example, CSA, CSB, and RNA polymerase II [70]. XAB2 contains 15 TPR (tetratricopeptide repeat) motifs and appears to have a role in transcription and pre-mRNA splicing [71]. Generally, XAB2 serves as a guardian of POLR2A (the largest catalytic subunit of RNA polymerase II) expression to ensure global gene expression and to antagonize cell senescence [72]. Furthermore, XAB2 promotes homologous recombination and facilitates histone acetylation events linked to homologous recombination [73]. Immunoprecipitation studies have revealed that XAB2 interacts with endonucleases ERCC1–XPF and XPG outside NER under the conditions that favor the formation of R-loops [74]. Altogether, XAB2 is involved in R-loop removal and pre-mRNA splicing; both processes are linked to transcription [75]. Future experiments will shed light on the possible involvement of XPA in processes where XAB2 is active.

## 3. The Hinge of the NER Complex

DNA damage can be recognized by NER in one of two subpathways, which overlap except for the mode of DNA damage recognition. GG-NER can search for damage anywhere in the genome throughout the cell cycle [76]. TC-NER is responsible for the accelerated repair of lesions in the template DNA strand of actively transcribed genes only. In general, a good NER substrate should be bulky and must destabilize a DNA double helix (i.e., disrupt the base pairing and bend the duplex). To detect both conditions, NER has evolved special bipartite substrate discrimination: firstly, it recognizes a local thermodynamically destabilized site, and then, the latter is probed for the presence of a lesion.

**Figure 3:**
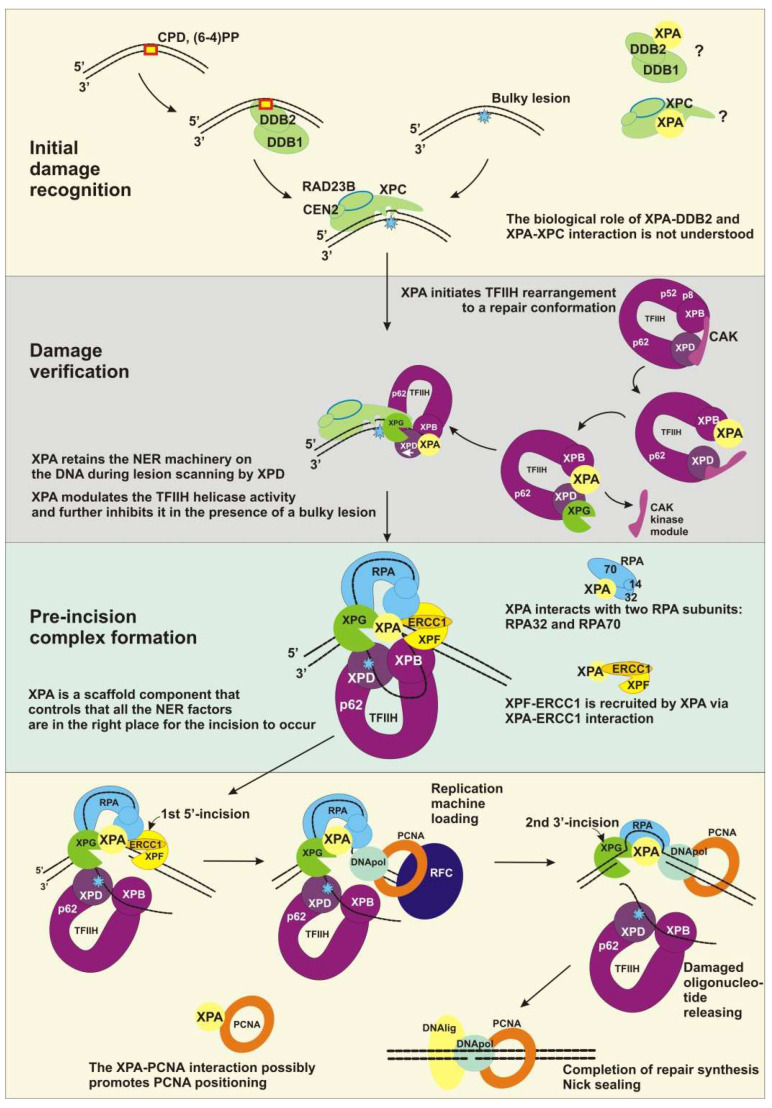
XPA organizes GG-NER machinery functioning. (Initial damage recognition) XPA interacts physically with DDB2 and XPC, but the biological role of these interactions is not understood. (Damage verification) XPC recruits TFIIH through XPC–p62 interaction. When XPA joins this complex through interaction with p8 and p52, it induces TFIIH rearrangement through binding XPB. During XPB binding, XPA engages the Cen2-binding site on XPB and possibly promotes XPC complex displacing. Next, XPG joins the complex by occupying the CAK-binding site on XPD. Thus, XPA and XPG promote CAK releasing and XPA “turning on” TFIIH repair conformation. During the damage verification, XPA retains XPB on DNA (see also Figure 4), modulates XPD helicase activity, and further inhibits it in the presence of a bulky lesion. (Pre-incision complex) XPA drives RPA binding to the undamaged strand through XPA–RPA32 interaction and stabilizes the PIC interior by additional interaction with RPA70. PIC becomes completed after XPF–ERCC1 engagement, which is recruited by XPA. XPA is a central component in the PIC, which makes sure that all the NER factors are in the right place for the incision to occur. At the later stages, XPA remains in the NER complex via a protein–protein interaction with the RPA that stays bound to the undamaged strand. Additionally, XPA possibly promotes the positioning of the PCNA clamp.

In the case of mammalian GG-NER, lesions are recognized by the XPC–RAD23B–CEN2 complex (hereinafter, XPC) [3] (Figure 3). The XPC complex can detect and bind DNA sites where a regular double-helical structure is perturbed, and as a result, one or more base pairs are disrupted and/or destabilized [7,77,78,79,80]. The major UV-induced photoproducts CPD and 6-4PP are recognized by the DDB1/2 heterodimeric protein, which binds directly to these lesions and introduces a kink and an opening into the duplex, creates a more suitable substrate for XPC, and hands over the damaged substrate to the XPC complex. Next, XPC recruits TFIIH to the lesion site [2,81,82]. The PARP1 protein supports these NER events by chromatin de-condensation and by the poly(ADP-ribosyl)ation (PARylation) of NER participants, thereby facilitating their recruitment to the damage site [66,83]. TC-NER is initiated by the stalling of elongating RNA polymerase II (RNAPII) at DNA lesions [84,85]. Then, after its lesion stalling and assembly of factors CSB, CSA, and UVSSA, the latter promotes TFIIH binding downstream of RNAPII [86].

**Figure 4:**
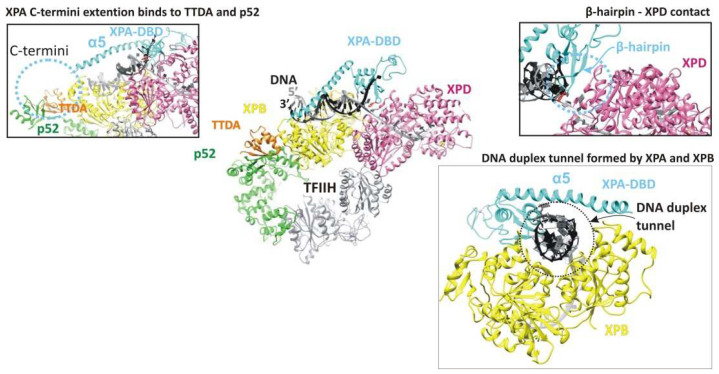
Cryo-EM structure of the human core TFIIH–XPA–DNA complex (PDB ID: 6RO4). XPA is colored light blue. The TFIIH subunits that are directly involved in the interaction with XPA: XPB, XPD, p52, and TTDA/p8 are colored yellow, magenta, green, and orange, respectively. In this complex, XPA forms a bridge between XPB and XPD, and XPA’s extended α5 helix and XPB form a positively charged tunnel that holds the DNA duplex within. XPA binds to XPD via its intercalating β-hairpin. The C-terminal region of XPA should be extended to p52 and TTDA/p8 and bind to these subunits, but unfortunately, it was observed at a lower resolution and was not used for the final model building (for details, please, see original research article [21]). The structures were generated using UCSF Chimera software (version 1.16).

The TFIIH complex is the key protein for the damage verification step [87]. TFIIH probes the lesion itself and unwinds the DNA duplex around the lesion, thereby making room for the subsequent assembly of repair machinery. Before TFIIH starts acting as a repair factor, it should be restructured from a transcription conformation to a repair one [21]. Both XPB and XPD bind DNA in the NER process, whereas only XPB binds DNA in transcription complexes [88,89]. During transcription, the CAK kinase module inhibits the repair XPD helicase activity via stabilization of the conformation where the XPD arch domain forms a “plug” structure that occupies the DNA-binding pore [21,90,91,92]. XPA (together with XPG) associates with TFIIH, releases the inhibitory CAK kinase module of TFIIH, and displaces the “plug” element from the DNA-binding pore in XPD [21] (Figure 3). This alternative TFIIH conformation results in the binding of XPB and XPD to the duplex and single-stranded 3′-DNA extension, consistently with their translocase and helicase activities, respectively (Figure 4). XPA forms an elongated arch over the DNA that bridges between the two ATPases. XPA binds to XPB and XPD via its extended α5-helix and its intercalating β4–β5 hairpin, respectively. The C-terminal region of XPA extends to p52 and TTDA/p8; this can explain why TTDA/p8 facilitates XPA recruitment in vivo. XPA’s extended α5-helix, together with ATPase XPB, forms a positively charged DNA duplex tunnel holding the DNA duplex within (Figure 4). Thus, XPA may keep NER machinery on the DNA during lesion scanning and processing. XPA chaperons TFIIH–DNA interactions and marks the DNA at the 5′ edge of the repair bubble [21]. Notably, at what moment XPA associates with TFIIH (or what signal promotes this association) is still unknown.

TFIIH’s structural flexibility allows XPD to unwind DNA while tracking along in the 5′→3′ direction [2]. During the tracking process, XPD pulls the DNA through a narrow tunnel that is too small for bulky DNA lesions to pass through. This “damage filtration” process is simple but effective.

Immediately after the formation of an undamaged ssDNA inside the repair bubble, this strand is covered by the RPA protein [48,93]. RPA protects the undamaged strand from a nuclease attack and additionally stabilizes the opened DNA intermediate [22]. The size of the NER-excised fragment coincides with the maximal length of the ssDNA platform for RPA binding (~30 nt), but it is obvious that RPA binds to undamaged ssDNA being formed at the moment when it reaches the size of the minimal RPA-binding platform (8nt) [94]. RPA OB domains bind to ssDNA in a certain order in the 5′→3′ direction: firstly, RPA70A binds to ssDNA closer to the 5′ side, then RPA70B binds from a 3′ side of RPA70A, then RPA70C, and RPA32D binds to the 3′ -side of ssDNA [49,50,95]. XPA tightly interacts with RPA inside the repair bubble, and together, they ensure the correct orientation of XPG and XPF–ERCC1 inside the repair bubble [51]. The interaction site consists of two contacts: one between the XPA N terminus and RPA32D and the other forms between XPA’s ZnF subdomain and two RPA OB domains: RPA70AB. The contact with RPA32D is important for the initial association of XPA with RPA, while the interaction with the large RPA subunit is needed for structural shaping of the NER complex on the opened DNA [52]. The crystal structure of *Ustilago maydis* RPA revealed that ssDNA in a complex with RPA is also U-shaped; for this reason, the 5′ edge and 3′ edge of the repair bubble are pulled together [96]. Thus, XPA binds the 5′ edge of the repair bubble and could interact with RPA70AB located near the 3′ edge.

After XPD stalls on the lesion (a bulky lesion is “stacked” in the DNA-binding pore), XPG binds to the 3′ edge of the repair bubble. The formation of the NER pre-incision complex is completed by the engagement of XPF–ERCC1, which is recruited by XPA via the XPA–ERCC1 interaction and is properly positioned by RPA via the RPA–XPF interaction [53,54,55,56,57,97].

Accordingly, the interior of the NER pre-incision complex is as follows: TFIIH stalls at the lesion, RPA covers the undamaged opposite strand, XPA marks the 5′ edge of the repair bubble, XPG marks the 3′ edge of the repair bubble, and XPF–ERCC1 binds behind XPA (Figure 3). The latter is a central component in the pre-incision complex space, because it interacts with all its compartments.

The DNA incision is first carried out by XPF–ERCC1 on the 5′ side of the damage site [98]. The resultant DNA intermediate contains a long gap (~30 nt) with a free 3′-OH group, a long flap bearing a lesion, and an intact template. Next, replication machinery loads to start the repair synthesis [99]. The following are possible sets of replication proteins: “DNA polymerase δ/PCNA/RFC1–RFC–p66”, “DNA polymerase ε/CTF18/RFC”, or “DNA polymerase κ/ubiquitinated PCNA/XRCC1” [43]. Which set of replication factors is definitely recruited remains unknown, but generally, this depends on the status of the PCNA ubiquitination, as well as on the use of distinct clamp loader complexes or XRCC1. At this stage, XPA remains in the NER complex via a protein–protein interaction with the RPA that stays bound to the undamaged strand [39]. Moreover, XPA possibly promotes the positioning of the PCNA clamp [59], and RPA recruits clamp loader RFC [100,101,102].

Repair synthesis can proceed halfway through the gap [43]. Then, the XPG-made incision cleaves the flap [64,87] and leaves a 5′ phosphate that is utilized in the nick-sealing reaction carried out by DNA ligase I or by the DNA ligase IIIα–XRCC1 complex [103,104,105]. Now, NER is completed.

## 4. The Ways to Control XPA

All aspects of DNA damage response (DDR) signaling and DNA repair pathways (NER, base excision repair, mismatch repair, homologous recombination, and nonhomologous end joining) are very important for cell survival; therefore, these processes’ participants are controlled via transcription and are precisely tuned by PTMs. As far as the XPA protein is the keystone for the entire NER process, it is an essential node regulating repair capacity. The XPA amount is controlled at the transcriptional level by a molecular circadian clock (and in several other ways) and at the post-translational level by the ubiquitin–proteasome system. Today, it is known that the XPA activity is modified by several PTMs: phosphorylation, acetylation, and PARylation.

### 4.1. The XPA Protein Amount Control—A Balance between Production and Degradation

#### 4.1.1. The Amount of XPA Molecules

It has been shown that normal human fibroblasts WI38-VA and HeLa cells have ~150,000–200,000 XPA molecules per cell [106,107]. This amount is nearly equal to that of the most abundant human SSB analog: the RPA protein, which is present at ~200,000–250,000 molecules per cell [107,108,109]. Furthermore, the XPA molecules amount is greater than that of other NER factors: XPC—25,000 per HeLa cell [107], TFIIH—100,000 [107], XPG—80,000 [107], and XPF-ERCC1—100,000 molecules per cell [107]. A possible explanation of this abundance is that XPA takes part in another cellular process. We should mention here that subsequent research in the XPA field has revealed that the XPA amount is not constant and depends on several conditions; therefore, whether this amount (about 200,000 XPA molecules per cell) corresponds to a maximum or minimum protein level is unclear.

The question about the number of XPA molecules is directly connected to another one: whether XPA is a rate-limiting factor in NER. The answer to this question is especially valuable for NER inhibitors screening: Should these compounds be more effective at reducing the XPA protein level or at disrupting the XPA protein function? Some studies indicate that XPA is not a rate-limiting factor of NER [106,110], while others have revealed the opposite: the peak NER activity coincides with the maximum XPA protein levels [111,112,113]. In a normal human fibroblast line immortalized by telomerase overexpression (NHF-1), when XPA is downregulated to 60%, 10%, or 4% of its original value, the rates of repair of both 6-4PPs and CPDs are proportionally reduced [114]. Thus, the UV sensitivity of human cells is a linear function of the XPA concentration [115,116].

#### 4.1.2. The XPA Residence: The Cytoplasm or Nucleus?

Given that XPA contains a NLS signal, it is logical to propose that this sequence ensures that the protein is sorted into the nucleus. Indeed, the first information about the XPA residence indicated that XPA is permanently localized to the cell nucleus [110]. The subsequently obtained information has revealed that XPA nuclear transport is cell cycle-dependent [117] and DNA damage-induced [118,119]. According to these data, in G1-phase cells, most XPA molecules are located in the cytosol, and there is only a slight accumulation of XPA in the nucleus after UV irradiation. In the S phase, most XPA molecules are also located in the cytosol, but they are imported into the nucleus after UV treatment. XPA is predominantly located in the nucleus in the G2-phase cell population with or without UV irradiation. The discrepancy between the reports on XPA localization is possibly due to a difference in the experimental methodology [119,120].

It has been demonstrated that protein adaptors importin-α4 and importin-α7 directly interact with XPA’s NLS for transporting XPA through the nuclear pore complex [119]. The adaptor importin-α7 is involved in XPA nuclear import independently of the DNA damage; hence, it is possible that importin-α7 is mainly responsible for the nuclear import of XPA in the G2 phase and for the basal XPA level required in the nucleus throughout the cell cycle. At the same time, the importin-α4–mediated nuclear import is DNA damage-induced, and DNA damage checkpoint protein kinase ATR is involved in this regulation (we discuss it below). The NLS of XPA cannot be efficiently recognized by importin-α4 in the absence of UV irradiation, perhaps owing to the masking of the XPA NLS by the binding of other cytoplasmic factors.

Tumor suppressor protein p53 is a major downstream effector and a phosphorylation substrate in the ATR-mediated DDR. The p53 status of cells significantly influences the role of ATR in the regulation of DNA repair after UV or cisplatin damage. The XPA nuclear import is dependent on p53 transcriptional activity in p53^+/+^ cells and is much slower in p53^−/−^ cells, but the import still proceeds. Therefore, the damage-induced ATR activation of p53 appears to be a primary, but not the sole mediator of, XPA nuclear import in p53^+/+^ as compared to p53^−/−^ cells in the S phase [119,121,122,123].

The reason why, in the G2 phase, XPA stays sequestered in the cytosol, even with DNA damage, is unclear. Nonetheless, the cytoplasmic XPA localization is consistent with the information about XPA interacting with mainly cytoplasmic proteins XAB1 and XAB3–5 [69]. As mentioning above, data about the interactions with these novel proteins have been obtained using the yeast two-hybrid system. The role of these XPA interactions stays unclear. In a database search, XAB3 was identified as metallopeptidase PRSM1 [124], XAB5 is the Golgi reassembly stacking protein GRASP65 [125], and XAB4 has a region homologous to GRASP65.

XPA-binding protein 1 (XAB1) is a GTPase. The XAB1 interaction site (aa 30–34) coincides with the NLS of XPA (aa 30–42), and therefore, it could be suggested that XAB1 is involved in the nuclear localization of XPA or interferes with the system of XPA nuclear transport [69]. Nevertheless, a small-interfering RNA (siRNA) knockdown of XAB1 has no effect on the UV-induced nuclear import of XPA [119]. This contradiction could be due to the different environments within human cells and the yeast model system. A GTPase other than XAB1 must be involved in the nuclear import of XPA in human cells.

#### 4.1.3. The Circadian Rhythm of XPA’s Life

Most of living things on Earth have adapted their life to its 24-h light/dark cycle and have developed an internal oscillating system to sense the light–dark cycles: the “circadian clock system”. In mammals, the circadian clock is “ticking” not only at the organismal level by synchronizing the rhythmicity of sleep/activity cycles and behavioral changes corresponding to light and temperature cycles but also in every single cell to synchronize its biochemical reactions so as to respond to organismal energetic demands [126].

**Figure 5:**
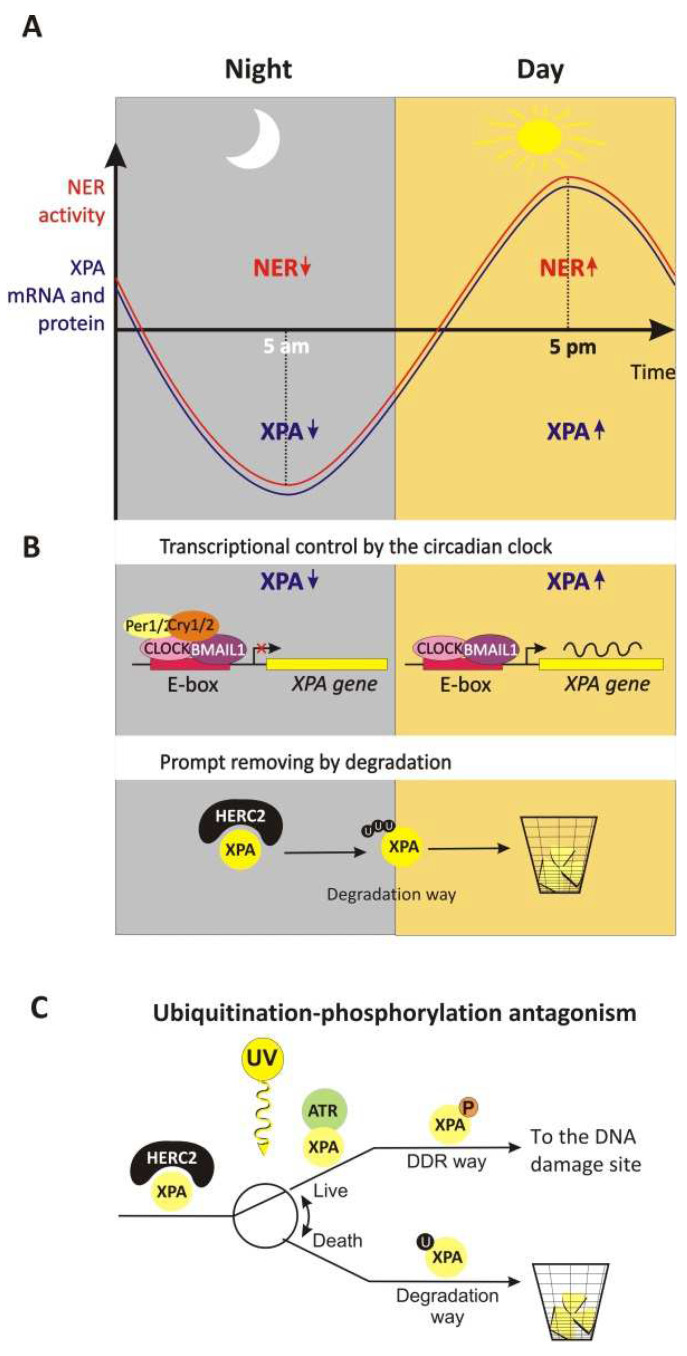
The circadian rhythm of XPA’s life. (**A**) The circadian rhythm of NER activity is due to the circadian oscillation of XPA mRNA and the protein levels. In a mouse model, it was shown for the brain, skin, and liver that the elimination of cisplatin–DNA adducts by the NER system exhibits a robust circadian rhythm, with the zenith in the late afternoon hours (~5 p.m.) and the nadir in the early morning hours (~5 a.m.). This oscillation is caused by the circadian rhythmicity of *Xpa* transcription and translation. (**B**) The balance between production and degradation. The mammalian circadian clock is generated by a transcriptional–translational feedback loop: core clock proteins CLOCK and BMAL1 activate the transcription of many clock-controlled genes, including repressor genes period (*PER1/2*) and cryptochrome (*CRY1/2*), by binding to E-box elements in their promoters and activating their transcription. After a time delay, the CRY and PER proteins accumulate in the cytoplasm, then form the CRY–PER complex, and are translocated back into the nucleus to inhibit their own transcription, as well as the transcription of clock-controlled output genes, through the inhibition of CLOCK–BMAL1 activity. The amount of repressor proteins CRY and PER is also regulated by secondary feedback loops at the transcriptional level, as well as by proteolytic degradation [126]. Accordingly, the XPA mRNA and protein levels are regulated positively by the CLOCK–BMAL1 complex and negatively by the CRY–PER complex. To attain a sufficient amplitude of the XPA protein oscillation level, circadian transcriptional regulation is coupled with the permanent prompt removal of the XPA protein by ubiquitin-dependent degradation. (**C**) UV light-induced damage and protective phosphorylation. In response to UV in a dose-dependent manner, ATR binds to and phosphorylates XPA. The XPA–ATR interaction facilitates HERC2 dissociation from the XPA complex, resulting in the accumulation of XPA molecules.

Since NER is the sole DNA repair mechanism capable of removing DNA photolesions in the skin [7], it seems obviously advantageous for organisms to have high levels of NER activity at the time of maximal exposure to sunlight within a day and to reduce NER activity at night, when it apparently is not needed [127]. Indeed, it has been shown that NER activity in mice is regulated by the circadian clock, such that repair activity changes by almost 10-fold over the course of the day, with the zenith in the late afternoon hours (~5 p.m.) and the nadir in the early morning hours (~5 a.m.) (Figure 5A) [111,112]. This regulation, which is accomplished through control over the XPA protein and NER activity oscillation, correlates with the XPA protein level circadian rhythmicity (in the mouse skin [113], in the mouse liver [112], and in the mouse brain [111]).

*XPA* gene transcription is regulated positively by the core circadian clock factors: circadian locomotor output cycles kaput (CLOCK) and its binding partner, brain and muscle ARNT-like protein 1 (BMAL1) [113]. The CLOCK–BMAL1 transcription activator complex binds DNA directly in regulatory elements (E-boxes) within rhythmicity-related genes to influence their transcription [126]. In our case, the CLOCK–BMAL1 complex binds directly to the promotor region of *XPA* [128] (Figure 5B). At the same time, major targets of CLOCK–BMAL1 include other core clock genes that encode the mammalian period ortholog (*PER1*, PER2, and *PER3*) and cryptochrome (*CRY1* and *CRY2*) repressor proteins [126]. These negative regulators heterodimerize and, then, after a time delay, translocate into the nucleus, where they repress their own gene transcription by inhibiting CLOCK–BMAL1. The time delay between the synthesis of Cry and Per and their actions as repressors generate an oscillatory pattern [111]. Thus, the Cry–Per complex forms a feedback loop ([129] and references therein) and negatively regulates *XPA* gene transcription [113].

Changing only the *XPA* gene transcription level may not be sufficient to produce the oscillatory mode of its protein amount profile. Indeed, the XPA protein is subjected to prompt removal by degradation: the XPA half-life is approximately 4 h in the absence of DNA damage [111,112]. A large protein (HECT- and RCC-like domain-containing protein; HERC2) is reported to ubiquitinate XPA, thereby leading to its degradation by the ubiquitin–proteasome system [114,130]. Thus, the relatively short XPA lifetime allows the transcription circadian rhythmicity to form the sufficient amplitude of oscillation of the XPA protein level.

Upon UV-induced DNA damage, XPA is phosphorylated [123]. XPA phosphorylation inhibits HERC2-catalyzed ubiquitination, protecting XPA from subsequent degradation, and induces the accumulation of XPA molecules (Figure 5C). The increase in the XPA protein amount causes a proportional increase in the rate of repair of CPDs and 6-4PPs [114,130]. It is worth noting that UvrA, the key damage recognition protein in bacterial NER, is also tightly regulated by proteolysis [131].

#### 4.1.4. XPA Transcription Control Inside a Solid Tumor

The oxygenation level is generally reduced and heterogeneous within a solid tumor as a consequence of the high tumor cell proliferation rate [132]. Hypoxic regions are thought to be present in about 50% of solid tumors: within most solid tumors, the oxygen level fluctuates between physioxia (~8% O_2_, i.e., 60 mmHg), hypoxia (~1% O_2_, i.e., 7.5 mmHg), and anoxia (0% O_2_) [133]. Unfortunately, hypoxia is associated with malignant progression, treatment resistance, metastasis, and poor prognosis [134]. Tumor cells adapt to low oxygen levels by inducing angiogenesis, increasing glucose consumption, and switching to glycolysis. Master regulators of the transcriptional response to hypoxia are transcription factors HIF (hypoxia-inducible factor), especially HIF-1. The regulation of the HIF-1 levels is very dynamic and adapts a tumor quickly to the oxygen concentration by inducing tumor angiogenesis (for the mechanism of HIFs’ regulation, see [134]).

It has been found that, under hypoxic conditions, subunit HIF1α binds with a strong affinity to a hypoxia response element in the *XPA* promoter and upregulates XPA expression approximately fivefold [135]. Modulating the expression of HIF1α by siRNA (downregulating *HIF1α* expression) or cobalt chloride (inducing hypoxia and HIF1α upregulation) markedly reduces or increases the transcription of *XPA* in lung cancer cell lines, respectively [135]. Consequently, the chemotherapy resistance related to hypoxia could be attributed not only to limited oxygen availability but also to the HIF1α-induced upregulation of XPA and subsequent NER activation.

Genes of several NER factors also contain putative hypoxia response elements in the promoter region (*XPB*, *XPC*, *XPG*, *CSA*, and *CSB*), suggesting that HIF-1α is a regulator of DNA repair machinery [136]. It is reported that the expression of XPD is upregulated by HIF-1α immediately after UV light exposure. In the case of XPC, the regulation is more complicated: immediately after UV irradiation, HIF-1α downregulates *XPC* expression, but after some delay, HIF-1α is phosphorylated and promotes the subsequent *XPC* expression increasing [136].

Nrf2 (NF-E2-related factor 2) is a transcription factor required for the response to reactive oxygen species or exposure to electrophiles activating the gene transcription of a set of drug-metabolizing enzymes [137]. Recently, it was shown that Nrf2 binds to an enhancer element in the *HIF-1α* gene, thereby promoting *HIF-1α* mRNA synthesis under mild hypoxic conditions (5% O_2_). As a result, Nrf2-dependent transcription counteracts HIF-1α degradation and leads to a preferential cisplatin resistance in hepatocellular carcinoma cells. Therefore, there is probably a Nrf2 influence on *XPA* expression by means of HIF-1α.

We should mention here that hypoxic conditions promote the formation of cyclopurines (8,5′-cyclopurine-2′-deoxynucleosides) and their accumulation. This specific class of endogenous oxidative DNA lesions is NER substrates and is currently considered the endogenous DNA lesions responsible for neurological problems in XP [7].

#### 4.1.5. Other Ways to Control XPA Transcription

High-mobility group proteins (HMGs) are nonhistone nuclear proteins binding nucleosomes and participating in all aspects of the chromatin structure and function, including DNA repair processes. HMGA family proteins have been demonstrated to preferentially bind to minor grooves of short fragments of A/T-rich DNA by recognizing structures rather than nucleotide sequences [138,139]. The overexpression of HMGA1 in MCF-7 breast cancer cells causes NER deficiency after UV light exposure [140]. Thereafter, it has been revealed that HMGA1 represses *XPA* transcription via binding to an A/T-rich negative regulatory element in the *XPA* promoter [141]. The XPA protein levels are also lower in HMGA1-overexpressing MCF-7 cells.

Numerous studies have indicated that C-type lectin domain family 4 member M (CLEC4M) is associated with the progression of various tumor types [142,143,144,145,146]. Moreover, a high *CLEC4M* expression level is implicated in cisplatin resistance and correlates with a poor prognosis in patients with lung cancer (non–small cell lung cancer) [147]. Further experiments have revealed that CLEC4M improves the NER capacity by raising the *XPA* and *ERCC1* mRNA levels. The mechanism of these regulatory actions is not well-understood, because CLEC4M is normally a transmembrane protein that helps to recognize a range of pathogens and mediates the endocytosis of ligands [148,149]. In addition, CLEC4M is able to promote cell migration with or without cisplatin treatment. Unfortunately, two subsequent simultaneously published reports on a hepatocellular carcinoma model yielded contradictory results: a high CLEC4M expression is associated with a poor prognosis [150] or with a favorable prognosis for the patients [151].

### 4.2. Fine Tuning of XPA by PTMs

#### 4.2.1. Phosphorylation and Checkpoint/DNA Repair Duties

In response to DNA damage, mammalian cells arrest cell cycle progression and activate the DDR mechanism. The DDR pathway is composed of four principal elements of (i) DNA damage sensors that directly recognize aberrant DNA structures and activate (ii) master transducers—DDR kinases; (iii) signal mediator proteins (which facilitate the phosphorylation events within the DDR network); and (iv) downstream effector molecules that are participants of a wide spectrum of cellular processes important for genomic stability, such as DNA replication, DNA repair, and cell cycle control [152,153].

**Figure 6:**
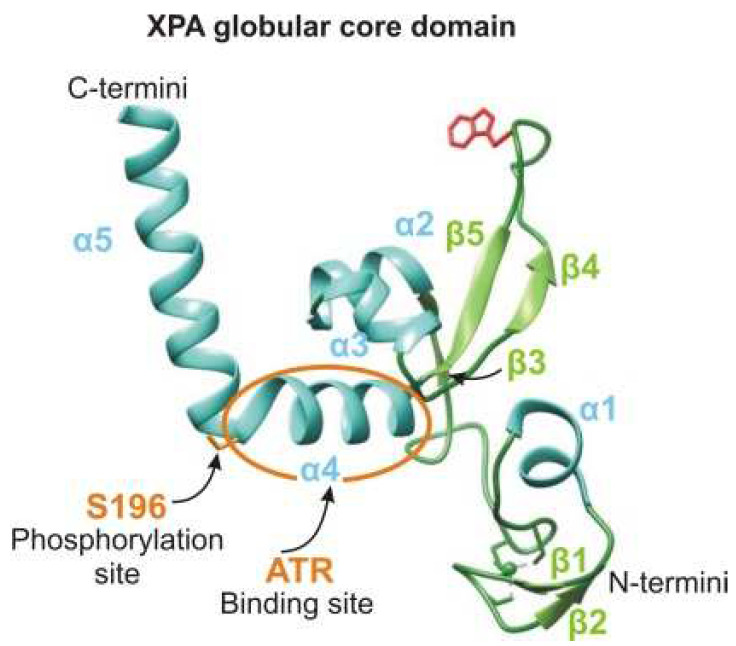
The ATR interaction site on XPA is the α4-helix located on the N-terminal side of the α4-helix-turn-α5-helix motif. ATR phosphorylates XPA at serine 196 (S196), which is located in the “turn” element inside the helix-turn-helix motif. The color codes correspond to Figure 1A. The structure was generated using UCSF Chimera software (version 1.16).

The checkpoint pathway mediated by the ATR (ataxia–telangiectasia and Rad3-related)–ATRIP (ATR-interacting protein) serine/threonine protein kinase complex plays a central role in the DDR and is mainly triggered by single-stranded breaks and base modifications, including the damage generated by UV irradiation [127]. In particular, the ATR–ATRIP kinase complex may uniquely participate in NER modulation through a direct XPA–ATR interaction [67,118,121]. The ATR interaction site on XPA is the α4-helix located on the N-terminal side of the helix-turn-helix motif of XPA’s DNA-binding site [67] (Figure 1A and Figure 6). The XPA Lys188 residue mediates this interaction but is not directly involved in it. It is possible that ATR is able to remodel its XPA-binding surface, and Lys188 modulates the stability of the α-helix.

The significance of the ATR–XPA interaction has been shown by means of its necessity for XPA nuclear import in response to the UV irradiation of cells [118]. In particular, ATR modulates XPA nuclear transport in a cell cycle-dependent manner. During the S phase, the importin-α4-mediated transport of XPA is activated by UV radiation (in S-phase cells without DNA damage, XPA is mostly cytosolic) and requires functional ATR kinase activity [119,123]. Nonetheless, abrogating the phosphorylation of XPA by ATR has no effect on XPA nuclear import [67]. This finding suggests that the dependence on XPA–importin-α4 binding on ATR may be mediated by ATR’s regulation of other cytoplasmic factors participating in the modulation of XPA–importin-α4 binding [119,122].

Obviously, ATR–XPA complex formation is also required for XPA phosphorylation by ATR. In response to UV in a dose-dependent manner, ATR phosphorylates XPA at serine 196 (S196), which is located in the “turn” element inside the helix-turn-helix motif of the minimal DBD [67] (Figure 1A and Figure 6). As mentioned above, UV-induced ATR–XPA interaction and XPA phosphorylation stimulate NER by facilitating HERC2 dissociation from the XPA–HERC2 complex, leading to the accumulation of XPA proteins [112,114,127,130] (Figure 5). Thereafter, the substitution of Ser196 with aspartic acid for mimicking the phosphorylation of XPA showed delayed degradation kinetics compared with wild-type XPA, owing to impairment of the association with HERC2, resulting in reduced ubiquitination of the S196D mutant and, hence, in an enhanced NER capacity and increased cell resistance to UV irradiation [118,127]. The disruption of this phosphorylation site in XPA by the S196A mutation leads to the persistent association of HERC2 with the XPA complex [130]. XPA-deficient cells complemented with the XPA-S196A mutant manifest significantly reduced the repair efficiency of CPDs but not of 6-4PPs [118]. The NER of 6-4PPs is generally much more efficient than the repair of CPDs [7], and the above finding indicates that XPA phosphorylation may play a role in the removal of persistent DNA lesions, such as CPD [123].

During S-phase progression in unperturbed cells, ATR monitors replication fork progression and is required to protect cells from intrinsic replication stress [152]. XPA phosphorylation occurs in response to replication fork stalling at later stages in lesion removal [118]. Phosphorylation enhances the chromatin retention of XPA and influences XPA’s association with protein partners. In particular, the XPA phosphorylation status affects its binding to RPA, and the S196A substitution decrease resulted in a decreased XPA affinity for RPA70 [130].

**Figure 7:**
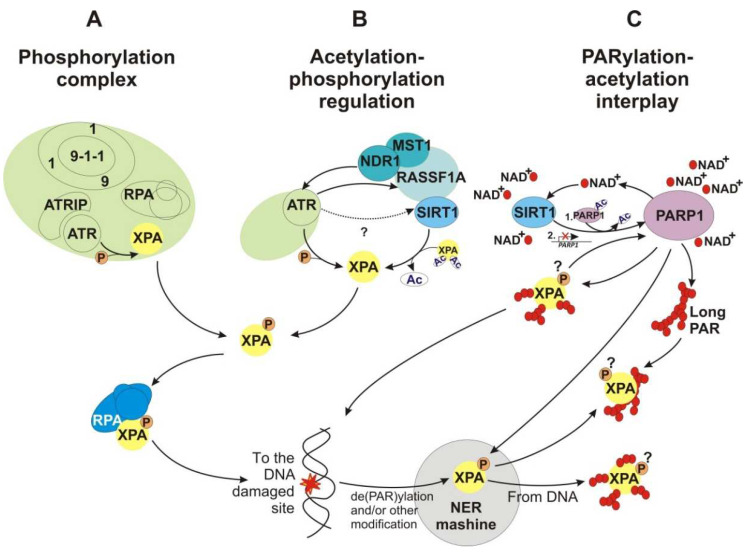
The network of reciprocal regulatory mechanisms of XPA’s PTMs. (**A**) The putative phosphorylation complex. ATR phosphorylates XPA at S196. (**B**) Acetylation–phosphorylation regulation. XPA is acetylated at Lys63 and Lys67. SIRT1 promotes NER by deacetylating XPA. The existence of an ATR–SIRT1 interaction is unclear and is indicated by the question mark. (**C**) PARylation–acetylation interplay. XPA is PARylated by PARP1. Both PARP1 and SIRT1 use NAD^+^ for their activity and interact physically. There is a strong connection between acetylation and PARylation. SIRT1 may deacetylase PARP1 and inhibit the PARP1 enzymatic activity. Under severe stress, PARP1 can become overactivated and may deplete cellular NAD^+^, thereby leading to the repression of SIRT1 activity and suppressing *SIRT1* transcription. To prevent this situation, SIRT1 is also capable of negatively regulating the expression of the *PARP1* gene. Moreover, XPA has a high affinity for long PAR polymers. Whether the PARylation events involve phosphorylated XPA is unclear and is denoted by the question mark.

ssDNA could arise within different pathways of DNA metabolism, i.e., at DNA damage sites and stalled replication forks, and this intermediate is immediately bound by the RPA protein [49,50,154,155]. The RPA–ssDNA platform also constitutes the key physiological signal that recruits the ATR–ATRIP master kinase complex via a RPA–ATRIP interaction [156]. Thereafter, the sequential recruitment of several protein factors organizes a signaling complex that potentiates ATR–ATRIP kinase activity [152]. According to these complicated protein combinations, we propose the existence of a functional complex that includes RPA, the 9–1–1 complex [154,157], ATR–ATRIP, and XPA (Figure 7) and drives XPA phosphorylation.

Taken together, all the protein factors that positively affect the assembly of the ATR–ATRIP signaling complex and subsequent ATR kinase activation positively influence XPA phosphorylation and NER activation. Even GG-NER protein sensors themselves—XPC and XPE—can activate the ATR pathway in the G1 phase by recruiting ATR kinase to DNA damage sites [104,158,159,160]. These also include centrosomal protein CEP164 and centrosomal duplication participant NDR1. CEP164 was initially identified as a centrosomal protein and then as a chromatin-binding mediator protein functioning in ATR-mediated checkpoint activation upon UV damage [161]. It has been demonstrated that a *Cep164* knockdown decreases survival and reduces the efficiency of CPD removal [68]. Upon UV irradiation, CEP164 interacts with XPA through aa 10–88 of XPA. The XPA–CEP164 interaction is essential for the localization of CEP164 to CPDs and for UV-induced cell cycle checkpoint kinase 1 (CHK1) phosphorylation. After DNA damage, ATR initiates a complicated signaling cascade via the phosphorylation of downstream protein substrates such as CHK1, whose activation leads to cell cycle arrest [127]. Thus, the CEP164–XPA interaction denotes a connection between DNA repair and checkpoint activation, especially at the G2–M checkpoint, where CEP164 plays a critical role [161]. A similar story applies to an XPA-interacting kinase called NDR1, which belongs to the nuclear-Dbf2-related (NDR) family of serine/threonine kinases functioning in processes related to cell cycle regulation, including centrosome duplication, apoptosis, and the alignment of mitotic chromosomes [162,163]. In the absence of DNA damage, NDR1 is mainly located in the cytoplasm. After UV irradiation, NDR1 colocalizes with XPA in the nucleus [163]. A siRNA knockdown of NDR1 delays CPD repair but does not affect 6-4PP repair (the result looks similar to the one obtained with XPA S196A mutation, see above). Instead, NDR1-depleted cells display a reduced activity of ATR toward some of its substrates, including CHK1 and p53, suggesting that NDR1 modulates NER indirectly via the ATR pathway.

Of note, another initially centrosomal protein is involved in the NER process: centrin 2 (human centrin 2, HsCen2, CEN2). In the nucleus, HsCen2 forms a stable XPC–RAD23B–CEN2 complex [164], influences the XPA–RPA binding to DNA [165], and even possesses an endonuclease-like activity [166].

#### 4.2.2. Dephosphorylation

Since activation of the DDR is mediated in part by phosphorylation, phosphatases are obvious candidates for the homeostatic regulators of DDRs. XPA dephosphorylation helps to switch off and remove the NER protein complexes after the repair process is completed. XPA is dephosphorylated by wild-type p53-induced phosphatase 1 (WIP1) [167]. This oncogenic phosphatase’s overexpression inactivates XPA and XPC and decreases NER activity; at the same time, WIP1-deficient mice show accelerated repair kinetics for UV lesions and less apoptosis.

Furthermore, WIP1 can inhibit the base excision repair, NER, and the double-stranded break repair proceeding via homologous recombination or nonhomologous end joining. Thus, the overall WIP1 role is to switch off DDR-activated proteins [167].

#### 4.2.3. Acetylation

Meanwhile, it has been reported that XPA is acetylated at Lys63 and Lys67 by an unknown acetyltransferase [168] (Figure 1A). In contrast to the positive phosphorylation effect, XPA acetylation works in a negative way and significantly reduces XPA function in NER. Target lysines Lys63 and Lys67 are located in the flexible disordered N-terminal region in the motif that is necessary for the interaction with ERCC1 and in close proximity to the RPA-binding domain (Figure 1A). Indeed, XPA acetylation significantly reduces its interaction with RPA. In particular, an acetylation-mimicking mutant (K6367Q) significantly reduces the interaction of XPA with RPA32, whereas XPA-deficient cells complemented with XPA-K6367Q have a significantly higher UV sensitivity. Similarly, when cells are treated with deacetylase inhibitors, this increases the wild-type XPA acetylation level, and the interaction with RPA32 is reduced [168].

It has been reported that XPA acetylation decreases NER activity [168], but there are contradictory data about the proportion of acetylated XPA: only 5% of acetylated XPA is observed in the mouse liver [114].

#### 4.2.4. Deacetylation

In mammalian cells, the histone deacetylase SIRT1 (belonging to NAD^+^-dependent class III of HDACs) is a crucial epigenetic regulator taking part in cell metabolism, genomic stability maintenance, reprograming, aging, and tumorigenesis [169]. In addition, SIRT1 favors NER by deacetylating XPA at residues Lys63 and Lys67 [168] (Figure 1A and Figure 7). UV light exposure promotes SIRT1–XPA complex formation, whereas SIRT1 downregulation significantly sensitizes cells to UV damage. The XPA-K6367R mutant, which mimics hypoacetylated XPA, rescues XPA-deficient cells upon UV treatment to the level seen with wild-type XPA. In contrast, XPA-K6367Q, an acetylation mimetic, results in a less efficient rescue upon UV treatment as compared to wild-type XPA. Furthermore, SIRT1-mediated XPA deacetylation enhances its interaction with RPA [168]. Given that the XPA phosphorylated state is necessary for RPA binding, it is reasonable to propose that this effect is due to the deacetylation presented protein surface for phosphorylation and promotes subsequent XPA–RPA complex formation.

It is known that ATM and ATR kinases often work together to send out a signal about DNA damage and regulate the downstream processes [152]. SIRT1 and ATM have a synergistic relationship, where SIRT1 is recruited to DNA breaks in an ATM-dependent manner, while SIRT1 also deacetylates ATM, and this stimulates its activity by autophosphorylation and stabilizes ATM at double-stranded break sites [170]. Although ATM is primarily activated by double-stranded breaks, ATR responds to a broad spectrum of DNA damages, including UV damage. Consequently, we could suggest, upon UV damage, SIRT1 is phosphorylated by ATR.

A two-hybrid screening has revealed a novel XPA-interacting partner: Ras association domain family member 1A (RASSF1A). This scaffold protein regulates the XPA acetylation–deacetylation cycle by recruiting the deacetylase SIRT1 to its substrate XPA upon UV exposure; thus, the XPA–RASSF1 interaction is essential for XPA repair activity [171,172]. It has been found that the RASSF1A A133S variant associates with several cancers [173], and at the same time, this variant inhibits XPA deacetylation, stabilizes the XPA–RASSF1 complex, and decreases DNA repair [171]. In response to a variety of DNA lesions, RASSF1A acts as a target of ATR/ATM-dependent phosphorylation [152,174]. Furthermore, the tumor suppressor RASSF1A protein is a key member of the RASSF1A–MST1 signaling cascade mediating apoptosis [175]. As mentioned above, XPA interacts with NDR1 kinase, whose kinase activity is activated in this pathway via the RASSF1A–MST1 interaction and triggers apoptosis. Thereafter, we suggest that the XPA protein is a key point between the DDR and apoptosis pathways. Moreover, according to the knowledge that NDR1 is a proximal regulator of the ATR pathway [163], it is possible to outline XPA’s putative network of reciprocal regulatory relations of acetylation/deacetylation and phosphorylation events (Figure 6).

In addition, we should mention that SIRT1 not only plays a NER role in XPA deacetylation but also catalyzes the deacetylation of RPA [176,177]. Additionally, SIRT1 enhances XPC expression [178,179].

Most chemotherapeutics’ anticancer activities are based on the induction of the DDR to promote the apoptotic pathway. Nonetheless, DNA repair pathways counteract this effect by repairing damaged DNA and restoring it to its normal status [180]. SIRT1 overexpression has been identified in several tumors [181]. Therefore, SIRT1-based NER upregulation is the mechanism that may underlie the development of cancer drug resistance [180].

During the manuscript preparation, we found that four articles from Dr. D’Orazio’s laboratory with Jarrett Stuart as the first author were retracted. That is why we did not discuss these articles, which were devoted to the XPA phosphorylation and acetylation.

#### 4.2.5. PARylation

PARylation by PARP1 (PAR polymerase 1) takes part in the PTM of the proteins involved in the various DNA repair pathways; modulates the proteins’ activity, localization, and turnover; and influences protein–DNA and protein–protein binding [83,182,183,184,185]. This modification is covalent and reversible [186]. On the other hand, the PAR polymer itself is widely accepted to be the third nucleic acid, and proteins can bind it noncovalently, just as DNA or RNA, via specific PAR-binding motifs [187,188].

It is reported that endogenous PARP1 is recruited to sites of local UV damage [189,190,191]. On the other hand, XPA has been found to be PARylated rapidly after UV radiation, and this modification facilitates XPA recruitment to a site of DNA damage [192,193] (Figure 1A and Figure 7). Which residues of XPA are PARylated has not yet been determined. According to the finding that XPA PARylation impairs its DNA-binding activity [193], we propose that the modified residues are located in the DBD or in close proximity to it. Therefore, how can we combine the low binding affinity of PARylated XPA with its better recruitment to the DNA damage site? Perhaps in close proximity to damaged DNA, XPA is de-PARylated.

The XPA protein contains a conserved PAR-binding motif within the C-terminal region mapped to residues 213–237 [193,194] (Figure 1A). This region consists of a consensus sequence of basic and hydrophobic residues and, interestingly, overlaps with the DDB2-binding and TFIIH-binding domains [41,47]. Thus, we propose that PAR binding modulates XPA interactions with these proteins and, furthermore, influences (or even manages) the XPA involvement in NER. The XPA affinity to long PAR chains (~55-mers) is much higher than that for short ones (~16-mers) and is in the nanomolar range [195].

Recently, it was reported that PAR molecules initiate the formation of nonmembrane compartments on the damaged DNA. In such compartments, the recruitment of DNA repair proteins is facilitated, while DNA damages and repair proteins are concentrated. Compartments have been shown to form in the presence of RNA-binding protein FUS, and its low-complexity domains are required for the compartmentalization [196]. Since both the N- and C-terminal parts of XPA are disordered, it can be hypothesized that XPA PARylation can initiate the assembly of the compartments that facilitate the NER process.

In addition, XPA physically interacts with PARP1 and further stimulates PARP1 enzymatic activity. The PARylation may strengthen the XPA–PARP1 interaction and promote additional PARylation events. This mutual influence combines with PARylation and is essential for the opening of the chromatin structure and proficient NER [192,193].

Both PARP1 and SIRT1 use NAD^+^ for their activity and interact physically. Moreover, there is a strong connection between acetylation and PARylation. Under stressful conditions, PARP1 is acetylated, and this modification enhances its enzymatic activity. Nonetheless, after that, SIRT1 may deacetylate PARP1 and inhibit PARP1 enzymatic activity. Under severe stress, PARP1 can become overactivated and may deplete cellular NAD^+^, thereby leading to repression of the SIRT1 activity [197,198] and suppressing *SIRT1* transcription [199]. To prevent this situation, SIRT1 is also capable of negatively regulating the expression of the *PARP1* gene [200]. If we add XPA, which is a target of both PARP1-induced and SIRT1-induced modifications, to this sophisticated picture of reciprocal regulatory relations, we will see another link between DNA repair and the stress response [7,201].

Aside from the XPA modification, PARP1 PARylates other NER protein participants: RPA [202], CSB [203], XPC [190], and DDB2 [204]. PARP1 and DDB2 can simultaneously bind to a CPD lesion in vitro [189]. PARP1 interacts with DDB2 and facilitates DNA damage recognition; at the same time, DDB2, just as XPA, stimulates PARP1 activity [183]. PARP1 also forms a stable complex with XPC and rapidly transfers this NER factor to DNA lesions in a DDB2-independent manner and can regulate XPC release [190,205]. PARP1 inhibition leads to the acceleration of DDB2 degradation and reduces XPC recruitment to UV lesions [183,204]. Moreover, the tight interaction between proteins XPC and PARP1 results in XPC-dependent stimulation of the PAR synthesis activity of PARP1 at UV-induced lesions, which facilitates the recruitment of a PAR-dependent chromatin remodeler called ALC1 [42]. XPA interactions with XPC and DDB2 contradict the widely accepted NER sequence of events, because XPA functions downstream of the initial damage recognition. A possible explanation of these interactions is that XPA takes part in chromatin remodeling.

Thus, we propose that (i) PARylation’s modification functional role is to promote XPA recruitment to a DNA damage site; (ii) the XPA–PARP1 interaction stimulates the PARP1 enzymatic activity that is possibly necessary for chromatin remodeling and for recruitment and the PARylation of other NER participants; and (iii) long PAR polymers could sequester XPA and increase its local concentration.

To summarize the described PTM data, we see a sophisticated network of modifications that—as a fine-tuning mechanism—regulate XPA localization, activity, and turnover (Figure 7). All of these modification reactions are regulated by each other in a reciprocal manner, thus raising several questions about how these mutual influences could take place: whether the reason is organized compartments with multiprotein complexes or maybe the modification events occur sequentially, and at each step, the enzymes validate and correct the upstream modifications.

## 5. Concluding Remarks

Nowadays, the data indicate that NER is a key predictor of the success of cisplatin chemotherapy [206,207]. At the same time, the NER capacity is a function of the XPA protein levels; therefore, knowledge about the ways of XPA control may find practical applications, such as the manipulation of NER repair activity for successful cancer treatment. Furthermore, structural information about the XPA protein–partner’s interaction surface organization is indispensable for targeted inhibitors modeling.

Additionally, the XPA protein level (same as the amounts of proteins that control it) may be a predictor of tumor sensitivity to cisplatin treatment [180]. In ovarian cancer, XPA has been shown to be expressed at a high level in tumors of patients resistant to cisplatin treatment [208]. XPA is expressed at low levels in testicular cancer, which is generally very responsive to cisplatin, thereby providing further correlative evidence for the importance of NER in cisplatin resistance [209,210].

A reduction in the NER capacity may be beneficial for patients with cancer who undergo chemotherapy, because this approach may allow reducing the dose of a DNA damage-inducing drug without compromising its therapeutic efficacy. From another point of view, it will be prominent to know the circadian rhythm in a healthy tissue to administrate the drug during the maximal NER activity to avoid side effects of the drug (cancer tissues are mainly arrhythmic); this approach is called chronotherapy [111,211]. Taking into account the circadian clock in the design of chemotherapeutic regimens will improve cancer chemotherapy by cisplatin and other drugs that produce DNA base damage repairable by NER.
